# A Satellite-Based Spatio-Temporal Machine Learning Model to Reconstruct Daily PM_2.5_ Concentrations across Great Britain

**DOI:** 10.3390/rs12223803

**Published:** 2020-11-20

**Authors:** Rochelle Schneider, Ana M. Vicedo-Cabrera, Francesco Sera, Pierre Masselot, Massimo Stafoggia, Kees de Hoogh, Itai Kloog, Stefan Reis, Massimo Vieno, Antonio Gasparrini

**Affiliations:** 1 Department of Public Health, Environments and Society, London School of Hygiene & Tropical Medicine, London WC1H 9SH, UK; 2 The Centre on Climate Change and Planetary Health, London School of Hygiene & Tropical Medicine, London WC1H 9SH, UK; 3 European Centre for Medium-Range Weather Forecast (ECMWF), Shinfield Rd, Reading RG2 9AX, UK; 4 Institute of Social and Preventive Medicine, University of Bern, 3012 Bern, Switzerland; 5 Oeschger Center for Climate Change Research, University of Bern, 3012 Bern, Switzerland; 6 Department of Epidemiology, Lazio Regional Health Service, 00147 Rome, Italy; 7 Swiss Tropical and Public Health Institute, Socinstrasse 57, 4051 Basel, Switzerland; 8 University of Basel, Petersplatz 1, 4051 Basel, Switzerland; 9 Department of Geography and Environmental Development, Ben-Gurion University of the Negev, Beer Sheva P.O.B. 653, Israel; 10 UK Centre for Ecology & Hydrology, Bush Estate, Penicuik, Edinburgh, Midlothian EH26 0QB, UK; 11 Medical School, University of Exeter, Knowledge Spa, Truro TR1 3HD, UK; 12 Centre for Statistical Methodology, London School of Hygiene & Tropical Medicine, London WC1E 7HT, UK

**Keywords:** fine particulate matter, aerosol optical depth, satellite, reanalysis, machine learning, random forest

## Abstract

Epidemiological studies on the health effects of air pollution usually rely on measurements from fixed ground monitors, which provide limited spatio-temporal coverage. Data from satellites, reanalysis, and chemical transport models offer additional information used to reconstruct pollution concentrations at high spatio-temporal resolutions. This study aims to develop a multi-stage satellite-based machine learning model to estimate daily fine particulate matter (PM_2.5_) levels across Great Britain between 2008–2018. This high-resolution model consists of random forest (RF) algorithms applied in four stages. Stage-1 augments monitor-PM_2.5_ series using co-located PM_10_ measures. Stage-2 imputes missing satellite aerosol optical depth observations using atmospheric reanalysis models. Stage-3 integrates the output from previous stages with spatial and spatio-temporal variables to build a prediction model for PM_2.5_. Stage-4 applies Stage-3 models to estimate daily PM_2.5_ concentrations over a 1 km grid. The RF architecture performed well in all stages, with results from Stage-3 showing an average cross-validated R^2^ of 0.767 and minimal bias. The model performed better over the temporal scale when compared to the spatial component, but both presented good accuracy with an R^2^ of 0.795 and 0.658, respectively. These findings indicate that direct satellite observations must be integrated with other satellite-based products and geospatial variables to derive reliable estimates of air pollution exposure. The high spatio-temporal resolution and the relatively high precision allow these estimates (approximately 950 million points) to be used in epidemiological analyses to assess health risks associated with both short- and long-term exposure to PM_2.5_.

## 1 Introduction

The World Health Organization estimates in seven million global deaths associated with air pollution (both outdoor and household) every year, emphasising that exposure to particulate matter (PM) is among the greatest causes of concern [1]. Fine particles with an aerodynamic diameter smaller than 2.5 μm (PM_2.5_) can penetrate the human circulatory system through the lungs and provoke multiple adverse health outcomes, including mortality [2], hospital admissions [3], lung dysfunction [4], cardiovascular diseases [5], and allergic reactions [6]. Usually, epidemiological studies collect air quality (AQ) data from ground monitors to quantify both short-term and long-term PM_2.5_ exposure associated with acute and chronic health effects, respectively. The limitation in this health assessment approach is the lack of continuous temporal records of PM_2.5_ and the limited spatial distribution of the monitors. Great Britain is an example of countries with very limited spatio-temporal coverage of PM_2.5,_ whereby the monitoring network is densely located only in major cities and widespread measurements of PM_2.5_ only started from 2010.

Remote sensing observations of aerosol optical depth (AOD) obtained from satellites, which measures how much direct sunlight has been scattered and absorbed by aerosol particles suspended in the atmosphere, has recently been proposed as an alternative to measuring PM variability for epidemiological purposes [7]. However, while offering the advantage of global coverage and relatively high spatio-temporal resolution, the use of AOD for PM_2.5_ exposure assessments presents limitations, for instance the fact that it represents the total atmospheric column concentration of the aerosol rather than surface values [8]. Unsurprisingly, early studies based only on satellite-AOD achieved very low performances in predicting PM_2.5_ [9]. Recent studies have proposed more sophisticated approaches, combining AOD measures with information from other satellite products, reanalysis data, chemical transport models, and geospatial features to improve the prediction of PM_2.5_. Such studies used various analytical methods, including multiple linear regression [10], land-use regression [11,12], and mixed effect models [13–17]. The last development in this research area is represented by the application of machine learning (ML) algorithms, including various architectures such as random forests [18–22], neural networks [19,21], and gradient boosting [19,23,24]. These have demonstrated higher performances, linked with an ability to model any kind of predictor(s)-response association and to deal better with the potentially complex relationship between PM_2.5_, spatial, and spatio-temporal predictors [19,25].

The aim of this study is to develop and apply a multi-stage satellite-based ML model to estimate daily concentrations of PM_2.5_ over a 1 km grid across Great Britain in the period 2008–2018. The analysis is based on a dataset with synchronised information from various data sources, such as several remote sensing satellite products, multiple climate and atmospheric reanalysis databases, chemical transport models, and spatial and spatio-temporal variables. The model is assessed through measures of predictive performance, error, and bias, obtained through cross-validation.

## 2 Materials and Methods

### 2.1 Study Area and Period

Great Britain is an island with an extension of 229,462 km^2^ surrounded by the Atlantic Ocean, Irish Sea, North Sea, and the English Channel. It comprises the countries of England, Scotland, and Wales with a total population in 2018 of almost 65 million [26]. According to the Köppen climate classification, the United Kingdom (Great Britain and Northern Ireland) is defined as having a warm temperate climate, fully humid with a mostly warm summer (cold summer for some parts of Scotland and England) [27]. The study area included 234.429 1 km grid cells (containing a unique identification code, cell-ID) from the original 1 km Great Britain National Grid Squares [28] for a period between 1 January 2008 and 31 December 2018.

### 2.2 PM_2.5_ and PM_10_ Observed Data

**Figure 1:**
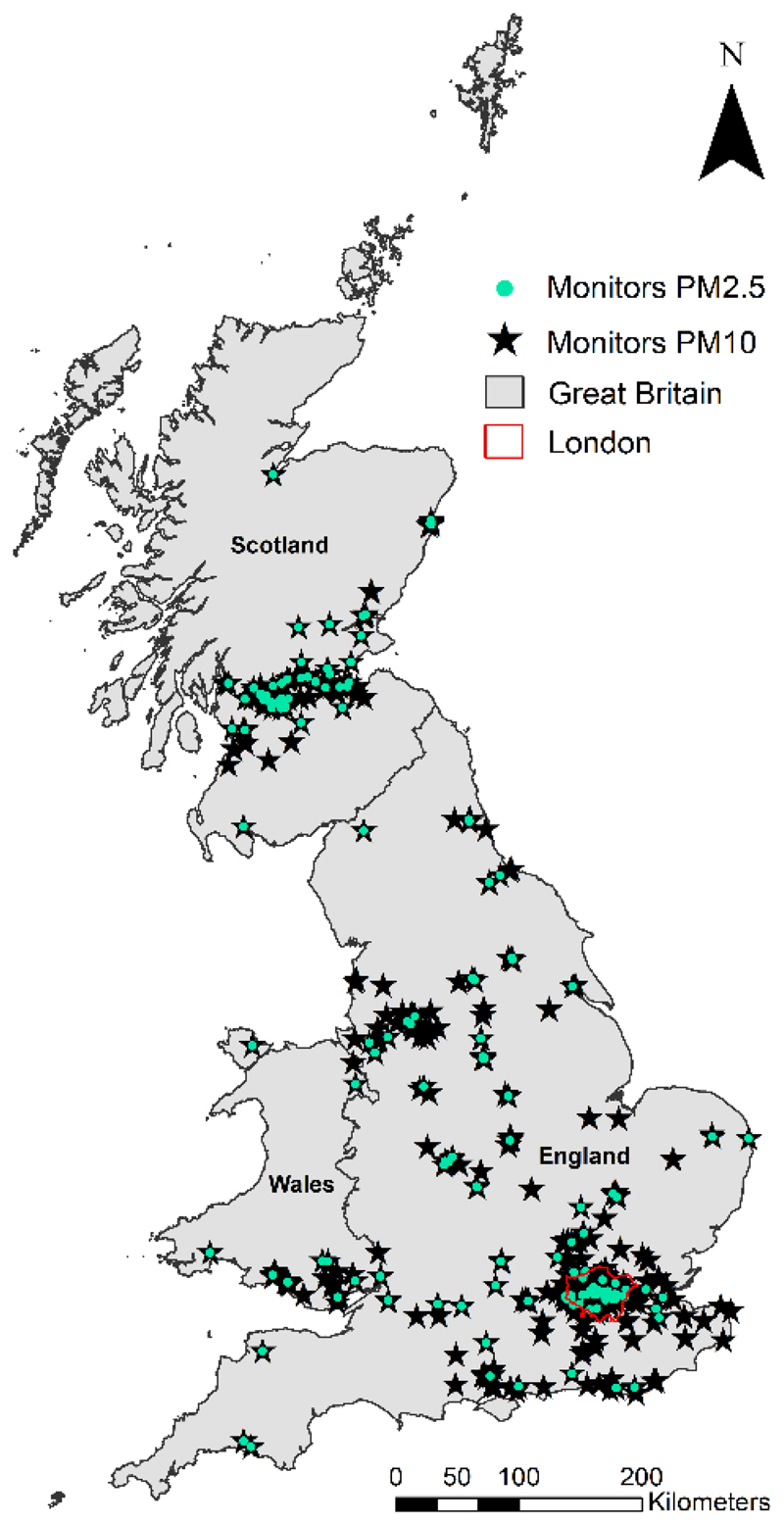
Spatial distribution of 581 PM_10_ (black star) and 183 PM_2.5_ (turquoise dots) monitors across Great Britain during the study period.

Daily PM_10_ and PM_2.5_ (μg/m^3^) measurements in the study period were obtained from five monitoring network sources through the R package openair [29]: Automatic Urban and Rural Network (AURN), Air Quality England (AQE), King College London (KCL), Scotland Air Quality Network (SAQN), and Wales Air Quality Network (WAQN). When monitors from different sources showed the same temporal distributions (i.e., a correlation equal to 1) and were located at approximately the same coordinates, only the AURN monitor was kept. Monitors with less than 18 hours of PM_2.5_ records per day as well as less than 30 days by year were removed. The final set includes 581 and 183 monitors measuring PM_10_ and PM_2.5_ along the study period, respectively. Figure 1 shows the locations of these monitors across Great Britain, illustrating how the network coverage is densely located in major cities, leaving several small cities and rural areas with only a few or no AQ records. Each monitor was indexed using the cell-ID of the 1 km grid cell that contained it.

### 2.3 Spatially-Lagged and Nearest Monitor PM_2.5_ Variables

Four spatio-temporal variables were generated from the monitor series of PM_2.5_ to represent spatially-lagged annual average concentrations. Monitor types were grouped into two classes (background (urban, suburban, and rural) and hotspots (traffic and industrial)) to compute the annual average values of nearby monitors by class using an inverse-distance weighted leave-one-out cross-validated (IDW-LOOCV) approach. Two different weights were applied, namely the inverse distance and the inverse squared distance (in km). The former assigns relatively more weight to distant monitors and therefore can represent a *regional* background, while the latter captures *local* differences. Therefore, these four IDW-LOOCV variables were named as follows: (i) Spatially-lagged hotspot-PM_2.5_ regional, (ii) Spatially-lagged background-PM_2.5_ regional, (iii) Spatially-lagged hotspot-PM_2.5_ local, and (iv) Spatially-lagged background-PM_2.5_ local. To improve the model performance in the spatial domain, two additional spatial variables were generated based on the closest Euclidean distance for each monitor class, named as: (i) Nearest hotspot monitor distance and (ii) Nearest background monitor distance. These six variables were used as additional predictors to capture the heterogeneity across monitors and exploit their spatial autocorrelation, and thus help the model to better categorise the differences in the spatial patterns of measured-PM_2.5_ series.

### 2.4 AOD Data: Satellite and Atmospheric Reanalysis Models

Daily satellite-AOD was obtained from the Collection 6 Level-2 gridded product (MCD19A2). These data are generated at a 1 km grid through the Multi-angle Implementation of Atmospheric Correction (MAIAC) algorithm using data from a Moderate Resolution Imaging Spectroradiometer (MODIS) sensor on board both Terra and Aqua Earth Observation satellites [30]. Four layers from the MCD19A2 product were extracted: (i) AOD Blue band (0.47 μm), (ii) AOD Green band (0.55 μm), (iii) AOD uncertainty (i.e., the level of uncertainty based on blue-band surface brightness (reflectance)), (iv) AOD_QA (quality assurance flags to retrieve only the best quality AOD). These layers are generated for each passing time of Terra and Aqua satellites over the area of study and combined by day. The layers AOD-0.47 μm and AOD-0.55 μm were used as the outcome variables after their values were filtered using AOD uncertainty and AOD_QA layers to guarantee high product quality.

This calibration process, together with pixels covered by clouds, removed a large sample of AOD grid cells over Great Britain. To fill the satellite-AOD gaps, the modelled-AOD total column was used from Copernicus Atmosphere Monitoring Service (CAMS) reanalysis provided by the European Centre for Medium-Range Weather Forecasts (ECMWF) [31]. CAMS reanalysis provides every 3-hourly modelled-AOD at five different wavelengths (0.47 μm, 0.55 μm, 0.67 μm, 0.87 μm, and 1.24 μm) with a spatial resolution of approximately 80 km, but the data were downloaded at 10 km, based on an interpolation performed through the ECMWF’s API request. The satellite-AOD and CAMS modelled-AOD were indexed to the closest 1 km grid cell from their pixel centroid.

### 2.5 Other Spatio-Temporal Predictors

#### 2.5.1 Modelled PM_2.5_ from Chemical Transport Models

Atmospheric chemistry transport models (ACTMs) incorporate anthropogenic and natural sources of emission, land use, and meteorological conditions to simulate the atmospheric compositions and deposition of various air pollutants (trace gases and particles). Based on the European Modelling and Evaluation Programme (EMEP) ACTM, EMEP4UK, has been developed to represent the UK hourly atmospheric composition at a spatial resolution of approximately 5 km [32]. The description of the EMEP4UK model framework and setup can be found elsewhere [33,34]. Daily EMEP4UK simulations of PM_2.5_ (μg/m^3^) concentrations at the surface-level were included to represent ground-level contributions, in contrast to AOD products that refer to the total column of aerosol concentration. Each EMEP4UK 5 km pixel was linked to the closest 1 km grid cell centroid.

#### 2.5.2 Meteorological Variables from Climate Reanalysis Models

Meteorological variables were retrieved from the ECMWF’s climate reanalysis models with the highest spatial resolution available during 2008–2018 and at two sub-day times (0:00 and 12:00). Sea-level pressure and the boundary layer height (BLH) were downloaded from the ERA 5 global reanalysis with a spatial resolution of approximately 30 km [35]. Air temperature at 2 m height and total precipitation were obtained from the ERA 5 Land global reanalysis with a spatial resolution of approximately 9 km [36]. Relative humidity, wind direction, and wind speed were downloaded from the UERRA regional reanalysis at 5.5 km for the MESCAN-SURFEX system [37]. All meteorological variables were indexed to the closest 1 km grid cell to their centroid.

#### 2.5.3 Normalized Difference Vegetation Index

Monthly Normalized difference vegetation index (NDVI) is used to quantify vegetation presence and it ranges from −1 to 1. The NDVI 1 km grid was obtained from MOD13A3 Version 6 Level 3, Terra-MODIS product [38]. Each NDVI pixel was indexed to the closest 1 km grid cell to its pixel centroid and the NDVI values repeated for the days inside the corresponded month.

### 2.6 Spatial Predictors

#### 2.6.1 Land Variables and Night-Time Light Data from Earth Observation Satellites

Three land predictors were collected from the Copernicus Land Monitoring Service (CLMS) [39] database: elevation, land cover, and impervious surfaces. Elevation data were obtained from the 2011 European Digital Elevation Model (EU-DEM) version 1.1 with a spatial resolution of 25 m. The elevation values were obtained from the mean of all 25 m-pixel values located inside each 1 km grid cell. Land cover data were obtained from the 2012 CORINE Land Cover (CLC) inventory. It was derived from high-resolution ortho-rectified satellites images that mapped all land elements at a spatial resolution ranging from 5 m to 60 m and aggregated into 100 m. Nine predictors were defined by grouping the original 44 CLC classes and each predictor represents its group proportion inside each 1 km Great Britain Grid cell. The imperviousness degree is a binary raster product at a spatial resolution of 100 m, where the value 0 represents natural land cover or water surface and value 1 represents entirely artificial surfaces (i.e., built-up areas). The amount of impervious surfaces was estimated by the proportion of artificial surfaces inside each 1 km Great Britain Grid cell.

Night-time lights data were provided by the visible Infrared Imaging Radiometer Suite (VIIRS) sensor aboard the Suomi-National Polar-orbiting Partnership (Suomi-NPP) satellite. The VIIRS Day/Night band collects cloud-free average radiance values at annual and monthly composites in a spatial resolution of 750 m [40]. The 2015 night-time lights annual mean was computed based on the weighted average of VIIRS pixels inside each 1 km Great Britain Grid cell.

#### 2.6.2 Population Density

The resident population counts data from 2011 that were collected from the Office for National Statistics (England and Wales) and the National Records of Scotland. The smallest geographic unit of the UK census is output areas (OA), with a total of 227.769 OAs polygons for Great Britain. The proportion of each OA’s area inside each 1 km Great Britain Grid cell was extracted to estimate the weighted average population by 1 km grid cell.

#### 2.6.3 Road Density and Distance

Road density and length predictors were derived from the Ordnance Survey (OS) [41] Open Roads product, which offers a geospatial representation of Great Britain’s Road network. Three density predictors were defined by grouping the original eight OS road types, where each predictor was computed as the sum of all roads length inside each 1 km Great Britain Grid cell by group (highway, secondary, and local). Three distance predictors were defined by computing the inverse distance of each 1 km grid centroid from the closest road group (highway, secondary, and local).

#### 2.6.4 Inverse Distance from Airports and Seashore

Information on location and size of airports was derived from the Civil Aviation Authority (CAA) [42], which collects monthly statistics about air traffic movements for more than 60 UK Airports, including aviation activities for terminal passengers, commercial flights, and cargo tonnage. A total of 19 airports across Great Britain were selected based on a minimum of 1% for the annual percentage of passengers at the airport from 2015–2018. For each cell of the 1 km Great Britain grid, the inverse distance from the closest airport was calculated.

The inverse distance from the seashore was computed for each 1 km grid cell using the geographical information about the boundaries of England, Wales, and Scotland provided by the UK Data Service [43].

### 2.7 Statistical Methods

A four-stage model was developed to obtain daily PM_2.5_ concentrations for all 234.429 grid cells covering Great Britain. Each stage is described in detail below. Briefly, Stage-1 applies a random forest (RF) algorithm to predict PM_2.5_ concentrations in monitors with only PM_10_ records. Stage-2 uses RF models to impute missing satellite-AOD from Terra and Aqua satellites, using modelled-AOD from CAMS. Stage-3 combines the output from Stage-1 and Stage-2 with a list of spatial and spatio-temporal synchronised predictors to estimate PM_2.5_ concentrations at the locations of the monitors. Stage-4 uses the Stage-3 model to predict daily PM_2.5_ across the whole of Great Britain.

#### 2.7.1 Random Forest Algorithm

RF is a supervised tree-based design ML algorithm that trains an ensemble of independent decision trees (or forests) in parallel. The final model accuracy is estimated by the performance average of all decision trees. There are two main advantages of the RF architecture (known as the bagging ensemble method): first, it controls the bias-variance trade-off by feeding each tree model with two-thirds of the training set while one-third is left out for validation (i.e., out-of-bag (OOB)) [44]. When all decision trees receive the same amount and list of predictors, they become highly correlated, not solving the variance problem. Therefore, the second positive aspect of RF is that the number of predictors on each tree is less than the full list available and they are randomly selected. This approach changes the predictor placed on the top of the tree, generating different splits and internal nodes. The algorithm is then able to estimate an importance ranking by quantifying the amount of error decreased due to a split of a specific predictor [45].

The performance of the models in each stage was assessed using statistics based on OOB samples and then from a 10-fold cross-validation (CV) procedure based on monitors. In the latter, ten random groups of monitors were defined, and the complete outcome series in each group were predicted using a model fitted in the other nine. This procedure offers a measure of the true predictive ability of the RF model in locations where no monitor is available. Measures of performance were generated by regressing the OOB or cross-validated predicted values on the observed series, and computing the R2, the root mean square error (RMSE), and the intercept and slope of the prediction. These statistics were computed overall using the whole series and then separated into spatial and temporal contributions. The former was computed using the averages of predicted and observed values across the series, and it offers a measure of performance in capturing long-term average PM_2.5_ concentrations. The latter was computed as daily deviations from the averages, and it quantifies the temporal variability explained by the model.

#### 2.7.2 Stage-1: Increasing PM_2.5_ Measurements Using Co-located PM_10_ Monitors

The number of PM_2.5_ monitors across Great Britain at the beginning of the study period was relatively low, and even if the quantity has increased substantially after 2010, most of the monitors were mostly installed in major cities. Stage-1 aims to increase the number of spatiotemporal ground-level PM_2.5_ references using observations from co-located monitors measuring PM_10_, which are available in a higher number of locations and are better distributed across Great Britain. Specifically, in this stage, we fitted an RF model in locations with both PM_2.5_ and PM_10_ measurements, using only data from the specific year-model (2008–2018). The RF model for each year *y* is defined as:

(1) $$PM_{2.5_{i,t}}^{y}=f\left(PM_{10_{i,t}},\;mon\text{.}type_{i},\;month_{t},dow_{t},\;{\text{lat}}_{i},\;{\text{lon}}_{i}\right)$$

where: $PM_{2.5_{i,t}}^{y}$ and *PM*_10_*i*,*t*__ are the target variable and main predictor, respectively, measured in year *y* at monitor *i* on day *t*; *mon*.*type*_*i*_ is a categorical variable classifying monitor *i* (traffic, industrial, urban, suburban, and rural); *month*_*t*_ and *dow*_*t*_ are categorical variables representing the months and day of the week of day *t*; and *lat*_*i*_ and *lon*_*i*_ define the coordinates of monitor *i*. The RF model was defined based on the best parameter setting. The optimised parameters were 500 decision trees as the RF ensemble (Ntree = 500) and 4 variables randomly selected to be used on each tree (mtry = 4). This model was eventually used to predict PM_2.5_ measurements in locations/days in which only PM_10_ was measured.

#### 2.7.3 Stage-2: Imputing Missing Satellite-AOD from CAMS Modelled-AOD

The percentage of missing satellite-AOD measurements in Great Britain ranged between 87% and 94% during 2008–2018 with the greatest portion during autumn and winter, near to the coast, and in the North of Scotland. Stage-2 imputes satellite-AOD missing for every day and 1 km grid based on an optimised RF model (Ntree = 50 and mtry = 20) and satellite-AOD wavelength (0.47 μm and 0.55 μm), separately in each year within the study period. These RF models were built for each year *y* as follows:

(2) $$\text{Satellite}-AOD_{i,t}^{\left(z,y\right)}=f\left(CAMS.AOD_{1,1_{i,t}},\ldots ,CAMS.AOD_{5,1_{i,t}},CAMS.AOD_{1,2_{i,t}},\ldots ,CAMS.AOD_{5,7_{i,t}},yday_{t},lat_{i},lon_{i}\right)$$

where: Satellite-$AOD_{i,t}^{z,y}$ is the target variable representing satellite-AOD (wavelength *z*, year *y*) estimates at grid cell *i* on day *t*; CAMS.AOD is the main predictor, representing CAMS modelled-AOD estimates at grid cell *i*, on day *t*, at five wavelengths (0.47 μm, 0.55 μm, 0.67 μm, 0.865 μm, and 1.24 μm), and at seven sub-day times (3 h, 6 h, 9 h, 12 h, 15 h, 18 h, and 21 h); *yday*_*t*_ defines the sequence of days in a year from 1 to 365 (366, for leap years); *lat*_*i*_ and *lon*_*i*_ represent the coordinates of grid cell centroid *i*. This model was eventually used to predict missing satellite-AOD measurements.

#### 2.7.4 Stage-3: Estimating PM_2.5_ Concentrations Using Spatial and Spatio-Temporal Variables

Stage-3 aims to build a predictive model for daily PM_2.5_ concentrations, using as target variable the combined set of PM_2.5_ directly measured from monitors or predicted from Stage-1, AOD measured from satellite instruments or predicted from Stage-2, together with all the spatially and spatiotemporally synchronized predictors described in the previous section. The RF models were fit separately by year *y*. The optimization process for this stage selected Ntree = 500 and mtry = 20 as the best set of parameter values. In this stage, PM_2.5_ concentrations were log-transformed to ensure the prediction of non-negative values. The Stage-3 model for each year *y* is defined as:

(3) $$\log\left(PM_{2.5}{}_{{}_{i,t}}^{y}\right)=f(SPT_{1_{i,t}},\ldots SPT_{15_{i,t}},SP_{1_{i}},\ldots ,SP_{27_{i}})$$

where: $\log\left(PM_{2.5}{}_{{}_{i,t}}^{y}\right)$ is the target variable representing the log-PM_2.5_ concentrations in year *y* at the monitor located in grid cell *i* on day *t*, while *SPT*_*i*,*t*_ and *SP*_*i*_ represent the spatiotemporal and spatial predictors, respectively.

Specifically, SPT_i,t_ included: surface-level log-PM_2.5_ values from the EMEP4UK model; Stage-2 AOD (at 0.47 μm, 0.55 μm); meteorological variables (air temperature, sea pressure, relative humidity, total precipitation, wind speed, and direction); BLH (at 12:00 and 24:00 hours); monthly-averaged NDVI; and day of the year, month, and week. SP_i_ included: spatially–lagged regional and local log-PM_2.5_ average concentrations from background and hotspot groups; nearest background and hotspot monitor distance; land variables (elevation, land cover in 9 groups, and % of impervious surface); night-time light; road density and inverse distance (each for highway, secondary, and local); population density; and inverse distance from closest airport and seashore.

#### 2.7.5 Stage-4: Reconstructing PM_2.5_ Time-Series at 1 km Grid

Using the RF models developed by year in Stage-3, daily PM_2.5_ concentrations for each 1 km grid cell were reconstructed across Great Britain for the whole study period (2008–2018).

## 3 Results

### 3.1 Stage-1 Results

Not all monitors in each network provided the full series of daily PM concentrations betwen 2008–2018. However, PM_10_ was more often available, especially in the years 2008–2009, when there were less than 80 PM_2.5_ monitors across Great Britain. The imputation process in Stage-1 enabled the expansion from 46 to 269 in 2008 and from 65 to 278 in 2009. Table 1 shows the Stage-1 model performance (i.e., a separate RF model for each year) reported by two CV methods: (i) OOB, the original RF CV algorithm, which presented better accuracy (R2 average of 0.932), and (ii) 10-Fold CV, a targeted sampling, which leaves out monitors with the full set of observations (R^2^ average of 0.855). As expected, the 10-Fold CV approach resulted in a slightly lower predictive accuracy although it still showed a good performance. There is some indication of bias in the 10-Fold CV, with a slope lower than the expected value of 1 and a slightly negative intercept. The results for 10-Fold CV spatial and temporal domains are in Table S1 in Supplementary Materials.

### 3.2 Stage-2 Results

**Figure 2:**
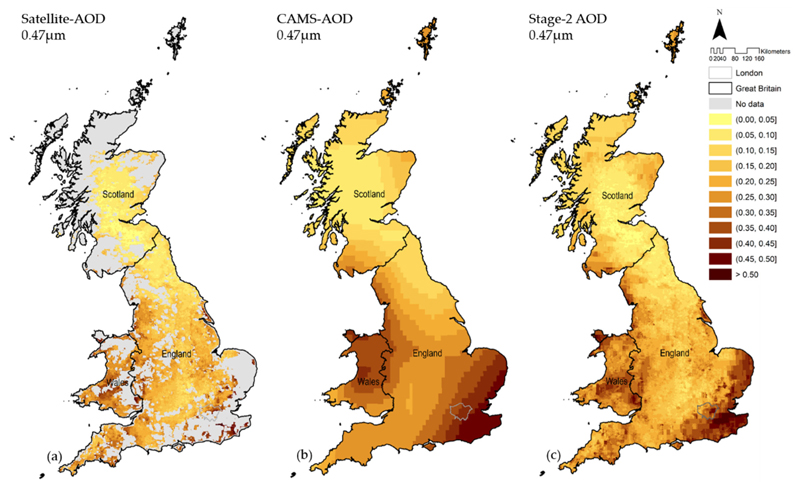
Satellite-AOD 0.47 μm values are represented in Figure 2a (mean of all Terra- and Aqua-MODIS passing times). The missing values in grey were imputed through a combination of multiple modelled-AOD wavelengths and sub-day times, represented in Figure 2b by modelled-AOD 0.47 μm at 12:00. The Stage-2 output is shown in Figure 2c, illustrating the full coverage of AOD 0.47 μm based on the combination of measurements and estimations across Great Britain. The maps correspond to values measured or reconstructed on 6 July 2018.

The Stage-2 procedure is illustrated in Figure 2, showing missing satellite-AOD, imputed through a combination of multiple modelled-AOD wavelengths and sub-day times. Table 2 shows the performance of Stage-2 models validated using the OOB CV method. The results presented consistently high R^2^ (ranging from 0.963 to 0.988), low RMSE (varying between 0.007 to 0.010), and almost no bias (intercept zero and slope close to 1 in almost all years and wavelengths).

### 3.3 Stage-3 Results

Stage-3, the main step of the satellite-based machine learning framework, combines the output of Stage-1 and Stage-2 with a list of spatial and spatiotemporal predictors to estimate PM_2.5_ at the locations of the monitors. The relative importance of the predictors for the Stage-3 RF models is ranked in Table 3. The list with the top-15 predictors demonstrates the larger contribution of the more informative spatiotemporal variables (EMEP4UK PM_2.5_, meteorological parameters, BLH, and sea-level pressure). All the proposed spatially-lagged PM_2.5_ variables were classified as highly important and their ranking positions varied slightly across the displayed years. This suggests the presence of spatial correlations in PM_2.5_ values that are not entirely captured by the other variables.

Table 4 shows the results of the 10-Fold CV Stage-3 RF models by year. The results indicate a good predictive performance of the model throughout the study period. Overall cross-validated R^2^ ranged from 0.704 (2008) to 0.821 (2011), with an average of 0.767. The average prediction error is 4.042 μg/m^3^, with a negligible bias in intercept and slope. The inspection of the spatial and temporal contributions shows that the model performs well generally across the two components, displaying a spatial performance drop only for 2008 (0.486) and 2015 (0.579). The cross-validated spatial R^2^ ranges from 0.486 (2008) to 0.746 (2017), while the cross-validated temporal R^2^ from 0.738 (2010) to 0.843 (2011). The two components have an average of 0.658 and 0.795, respectively. The high spatial R^2^ performance across the years demonstrates that the Stage-3 RF models were able to predict the spatial variation of long-term PM_2.5_ across Great Britain with good accuracy. In Supplementary Materials, Table S4 shows the Stage-3 results by season, demonstrating that the seasonal patterns were well described by RF models, although with a lower accuracy for the temporal domain in summer, characterized by a lower 10-Fold R2. This drop in performance during summer was also seen by other studies [17,20].

### 3.4 Stage-4 Results

**Figure 3:**
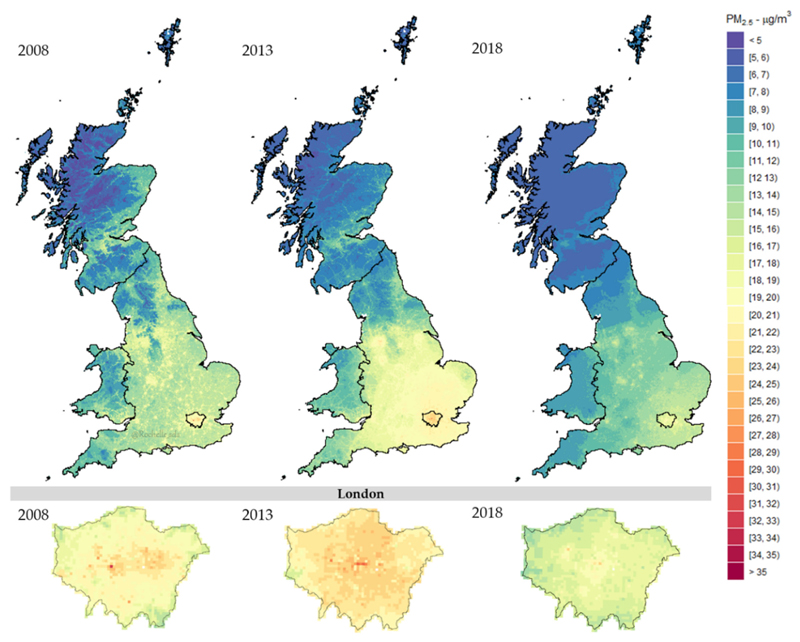
Stage-4 predicted PM_2.5_ concentrations across Great Britain (Top) and London (Bottom) for 2008, 2013, and 2018 aggregated by annual means. All plots were built under the same colour scale.

Stage-4 provides the prediction of daily PM_2.5_ concentrations for each of the 234.429 1 km grids covering Great Britain. Results indicate an annual PM_2.5_ average of 9.41 μg/m^3^ for 2008, 10.17 μg/m^3^ for 2013, 8.05 μg/m^3^ for 2018, and 8.84 μg/m^3^ for 2008–2018 but with a strong spatial and temporal variation. The spatial distribution of annual average PM_2.5_ concentrations for 2008, 2013, and 2018 are shown in Figure 3, revealing a decrease of pollution levels in recent years across the whole territory, although slightly stronger in England. Table S5 in Supplementary Materials provides the same figures for all the years, confirming the decreasing trend. The spatial comparison suggests that PM_2.5_ concentrations are lower in Scotland and Wales compared to the more populated southern regions of England, with hotspots located in urban areas such as Liverpool, Manchester, Birmingham, and Greater London. At the bottom of Figure 3, the maps display the corresponding annual average of PM_2.5_ levels in London, demonstrating the precision of the multi-stage ML model in reconstructing PM_2.5_ concentrations in 1 km grid cells within urban areas. The maps show local hotspots of high pollution, with a spatial distribution that, however, changes along the study period.

**Figure 4:**
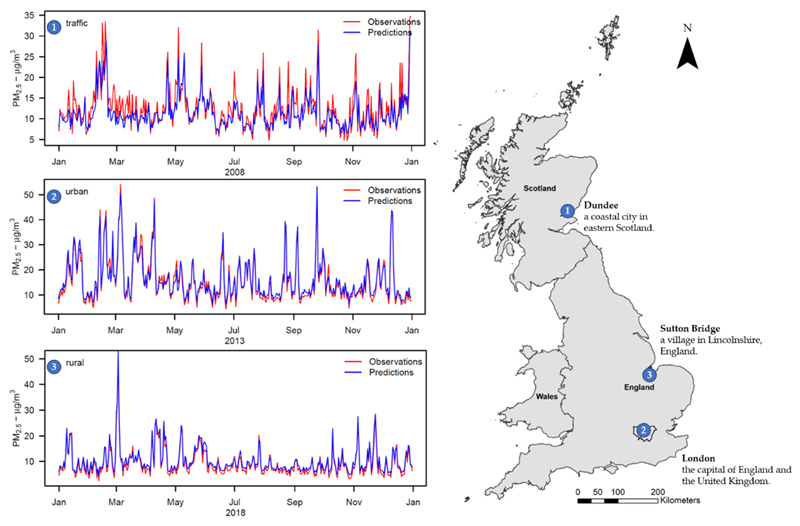
Stage-4 daily series of observed and predicted PM_2.5_ values across Great Britain for 2008, 2013, and 2018: (1) Traffic monitor—located in Dundee (Scotland); (2) Urban monitor—located in London (England); and (3) Rural monitor—located in Sutton Bridge (England).

The greatest contribution of this study is in the ability of the satellite-based ML models to reconstruct daily levels of PM_2.5_ over a 1 km grid across a wide geographical domain. Figure 4 displays the time series plots of observed and predicted PM_2.5_ concentrations at three monitoring sites and their locations within the geographical domain of Great Britain. While not necessarily representative, the results show that the error varies depending on location, type of monitor, and period. However, generally the plots indicate a very good performance of the ML algorithms in recovering the observed temporal variation in PM_2.5_ levels, capturing peaks and periods of stable low concentrations.

**Figure 5:**
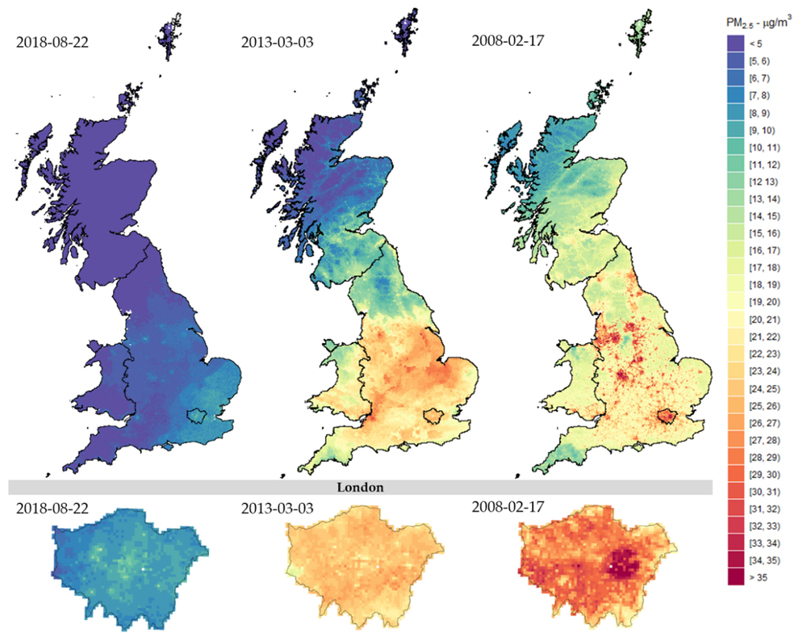
Stage-4 day-specific PM_2.5_ estimations across Great Britain (Top) and London (Bottom).

Figure 5 complements the analysis of daily variations by displaying the spatial distribution of PM_2.5_ estimations across Great Britain (Top) and London (Bottom) for specific days within the study period. It is interesting to note the wide variation in PM_2.5_ concentrations between days in the same area and between areas on different days. For instance, the maps in the left panels represent a day with almost no variation and generally low concentrations; the maps in the mid panels display a strong north–south split likely linked to weather conditions; the right panels show a more complex pattern with a wider range of PM_2.5_ values, a large area with very high pollution concentrations located in east London, and hotspots in highly populated urban areas across England.

## 4 Discussion

This study presents the first application of satellite-based spatiotemporal ML methods to reconstruct levels of pollution across Great Britain, providing estimates of daily PM_2.5_ concentrations over a 1 km grid during 2008–2018. The multi-stage ML framework provided significant advantages, allowing the combination of information from multiple data sources, such as air quality monitoring networks, remote sensing satellite products, chemical dispersion models, reanalysis databases, and administrative census data, among others.

The beginning of the study period (2008 and 2009) had a lower quantity of monitors measuring PM_2.5_ across Great Britain, but this number increased considerably from 2010 after a new measuring network had been established in 2009 [46]. Therefore, Stage-1 was an extremely important step to extend the number of PM_2.5_ measurements. The implementation of Stage-2 was also relevant to fill the gaps in MAIAC AOD retrievals from satellites, thus maximizing the available information. Stage-3 produced an ML prediction model based on a long list of informative predictors, accounting for potentially complex inter-relationships and functional forms. The Stage-3 ML algorithms offered excellent performance, showing an average cross-validated R^2^ of 0.767 across the period, with an increased predictive ability in the last years. Stage-4 provided a single PM_2.5_ estimation for each of the 234.429 1 km grid cells in each of the 4018 days, totalling around 950 million data points. The methodology provides complete spatial coverage, high resolution, and a relative small error of the predictions, and the ability to capture variations in PM_2.5_ concentrations across both spatial and temporal domains. The model offers a prediction accuracy that makes the output suitable for application in epidemiological studies on the short- and long-term health effects of air pollution. As demonstrated in Figure 4, the methodological approach presented in this study was able to capture the daily PM_2.5_ variability across different years, locations, and monitor types.

The output from the empirical ML model developed in this study complements existing databases of modelled PM_2.5_ in the United Kingdom, with some advantages for applications in epidemiological studies. Country-wide maps generated by emission-dispersion models are usually available at a coarser spatial or temporal resolution [32,47,48], and they generally show lower small-scale accuracy when tested against observed monitoring data [49,50]. The spatiotemporal ML models presented here demonstrated comparable predictive performance to similar methods applied in other countries, based either on single-learner ML models [13,20], ensemble ML models [18,19,21], or generalised additive models (GAM) [15]. Ref [21] developed an ensemble ML model (composed by RF, Deep Neural Networks, GAM, Gradient Boosting, K-nearest Neighbour) to estimate PM_2.5_ for Greater London, reaching a mean 2005–2013 CV spatial-R2 of 0.396. Using the 2008–2013 period, this study reached a CV spatial-R2 of 0.637 for the whole of Great Britain. Modelling a larger area might have provided more information and a higher spatial variability, improving substantially the CV spatial-R2. The‘application of a single learner to model air pollution in both spatial and temporal domains for Great Britain achieved a satisfactory performance. Nonetheless, ensemble model formats can be used in future applications using the same area of study and variables to assess how much performance gain is reached compared to the RF-only learner.

In 2008, a new Air Quality Directive came into force, bringing considerable changes to the following UK annual air quality assessments in 2010, setting an annual mean target of 25 μg/m^3^ [51]. Several policy controls and emission reductions had been put in place aiming to reduce from 2010 the road traffic PM emission by 83%, off-road mobile machinery by 54%, and energy production by 32% until 2020 [52]. Independently of the temporal aggregation (daily or annual), all maps shown in this study detected this considerable drop in the PM_2.5_ concentrations from 2010 across Great Britain.

Some limitations of this study must be acknowledged. First, the multi-stage model relies on the extension of the observed series of PM_2.5_ by predicting values based on co-located PM_10_ in Stage-1. This step was necessary for the application of the method in the early years characterized by sparse PM_2.5_ monitoring, which is likely to have contributed to the lower predictive performance in this period. Morover, the cross-validation procedures revealed the presence of some bias in the Stage-1 predictions, particularly in the spatial domain, which was probably linked to limitations in modelling the relative distribution of the two PM components. Second, while the model displayed a good performance throughout the period, the accuracy is worse in the temporal domain in the summer (Table S4), suggesting limitations in capturing the higher temporal variation in this season. Third, the generalization of the prediction model was dependent on the selected locations of the monitors, which may be not representative of the study domain. This can result in an underestimation of the error and potential biases in the predictions in more remote and less represented areas if structural spatial differences are not entirely captured by the model covariates.

The findings of this study also highlight additional limitations not yet discussed in previous studies related to the use of remote sensing satellite products for reconstructing air pollution exposure for epidemiological purposes. The RF importance ranking order showed that Stage-3 was mainly informed by EMEP4UK PM_2.5_, regional and local interpolated PM_2.5_ estimations, and meteorological variables, while the contribution from Stage-2 outputs (i.e., predicted-AOD 0.47 μm and 0.55 μm) was very limited. Therefore, direct satellite observations (i.e., AOD, NDVI, elevation, land cover, impervious surfaces, and night-time light) offered a relatively minor contribution to represent ground PM_2.5_ exposures (Stage-3), while satellite-based products (i.e., indirect satellite observations) such as EMEP4UK and climate reanalysis products played a much more relevant role to derive reliable PM_2.5_ estimates across Great Britain.

Future directions in remote sensing are pointing to new satellite instruments developed for air pollution monitoring which are likely to provide better resolution and reliability, thereby improving the predictive performance. As examples, the recently launched Copernicus Sentinel-5 Precursor [53] and the two future earth observation missions from European Space Agency (e.g., Copernicus Sentinel-4 [54] and Sentinel-5 [55]) were developed to provide information on atmospheric variables, such as air quality parameters (nitrogen dioxide, ozone, and aerosols). Finally, future research developments related to advanced statistical methodology, as demonstrated by the ML framework proposed here, include the application of geostatistical techniques, the use of alternative single-learner or ensemble ML algorithms, and statistical downscaling methods to increase further the resolution of the predictions.

## 5 Conclusions

This study developed and applied a multi-stage ML model, combining data for multiple sources, including remote sensing satellite products, climate and atmospheric reanalysis models, chemical transport models, and geospatial features, to generate a complete map of daily PM_2.5_ concentrations in a 1 km grid across Great Britain between 2008 and 2018. The model showed good performance overall and in both spatial and temporal domains, with an accuracy that is compatible with the use of such reconstructed values as a proxy for PM_2.5_ exposures in epidemiological studies. In particular, the availability of high-resolution measures that can be linked as such or aggregated at different spatial and/or temporal scales makes the output suitable for investigations on both transient and chronic health risks associated to short and long-term exposures to PM_2.5_, respectively.

## Supplementary Material

Appendix

## Figures and Tables

**Table 1:** Predicted-PM_2.5_ concentrations obtained from Stage-1 RF models were regressed against measured-PM_2.5_ concentrations in a linear regression model. The performance was evaluated using two CV methods (OOB and 10-Fold) together with RMSE (a measure of the model error, μg/m3), intercept (μg/m3), and slope (μg/m3).

Stage-1								
		OOB-CV				10-Fold CV		
	
	R2	RMSE	Inter.	Slope	R2	RMSE	Inter.	Slope
2008	0.918	1.196	–0.417	1.033	0.707	4.954	0.803	0.886
2009	0.921	1.110	–0.390	1.030	0.791	3.996	0.410	0.937
2010	0.919	1.122	–0.401	1.029	0.843	3.496	0.043	0.983
2011	0.949	1.124	–0.266	1.019	0.902	3.439	–0.087	0.997
2012	0.942	1.058	–0.274	1.021	0.889	3.218	–0.035	0.986
2013	0.929	1.087	–0.368	1.028	0.847	3.584	0.218	0.972
2014	0.944	0.963	–0.267	1.022	0.891	3.003	–0.007	0.995
2015	0.933	0.865	–0.265	1.026	0.871	2.662	–0.003	0.983
2016	0.935	0.896	–0.251	1.025	0.885	2.654	–0.050	0.996
2017	0.939	0.828	–0.196	1.022	0.895	2.430	0.010	0.985
2018	0.928	0.791	–0.248	1.028	0.886	2.235	–0.019	0.993
**Mean**	0.932	1.003	–0.304	1.026	0.855	3.243	0.117	0.974

**Table 2:** Predicted-AOD 0.47 μm and 0.55 .55 obtained from Stage-2 RF models were regressed against measured Satellite-AOD 0.47 μm and 0.55 μm in a linear regression model. The performance was evaluated using OOB CV together with RMSE (μg /m^3^), intercept (μg/m^3^), and slope (μg /m^3^).

**Stage-2 OOB CV**								
	Predicted-AOD 0.47 μm				Predicted-AOD 0.55 μm			

	R2	RMSE	Inter.	Slope	R2	RMSE	Inter.	Slope
2008	0.977	0.010	–0.001	1.009	0.977	0.007	–0.001	1.009
2009	0.976	0.010	–0.001	1.010	0.976	0.007	–0.001	1.010
2010	0.968	0.009	–0.001	1.013	0.968	0.007	–0.001	1.013
2011	0.988	0.010	–0.001	1.005	0.988	0.007	0.000	1.005
2012	0.980	0.010	–0.001	1.008	0.981	0.007	–0.001	1.008
2013	0.984	0.010	–0.001	1.007	0.984	0.007	–0.001	1 006
2014	0.970	0.009	–0.001	1.012	0.970	0.007	–0.001	1.012
2015	0.972	0.009	–0.001	1.011	0.973	0.007	–0.001	1.011
2016	0.975	0.009	–0.001	1.010	0.975	0.007	–0.001	1.010
2017	0.963	0.009	–0.001	1.015	0.963	0.007	–0.001	1.014
2018	0.969	0.010	–0.001	1.01113	0.969	0.007	–0.001	1.013
**Mean**	0.978	0.010	–0.001	1.009	0.9778	0.007	–0.001	1.009

**Table 3:** Relative importance (%) of the predictors in Stage-3 for the first, middle, and last years.

Stage-3 Predictors	2008	2013	2018
EMEP4UK PM_2.5_	32.41	32.83	36.74
Spatially-lagged hotspot-PM_2.5_ regional	2.55	2.49	6.77
Wind direction	6.35	7.33	5.34
Spatially-lagged background-PM_2.5_ regional	1.14	6.06	4.93
Day of the year	3.22	4.33	3.73
Spatially-lagged hotspot-PM_2.5_ local	3.97	1.65	3.66
Precipitation	6.63	2.42	3.25
BLH 0h	2.28	2.91	2.79
Spatially-lagged background-PM_2.5_ local	0.94	3.07	2.75
Month	1.76	2.72	2.68
2m Air temperature	2.60	2.93	2.65
Wind speed	3.08	3.75	2.53
Sea-level pressure	3.09	2.60	2.49
Relative humidity	1.77	1.56	1.81
Nearest non-traffic monitor distance	3.10	2.30	1.78

Note: The top 15 predictors in the RF importance ranking order is determined by the year 2018. The importance is measured by the amount of error reduced due to the splits of a given predictor over all trees used in the RF ensemble.

**Table 4:** Predicted-PM_2.5_ concentrations obtained from Stage-3 RF models were regressed against Stage-1 measured/predicted-PM_2.5_ concentrations in a linear regression model. The CV–R^2^ (how well the model described the PM_2.5_ variability in new locations) described in three different patterns (overall, spatial, and temporal), RMSE (μg/m^3^), intercept (μg/m^3^), and slope (μg/m^3^).

Stage-3												
		Overall				Spatial				Temporal		

	R2	RMSE	Inter.	Slope	R2	RMSE	Inter.	Slope	R2	RMSE	Inter.	Slope
2008	0.704	4.547	–1.251	1.064	0.486	2.698	–0.749	1.026	0.760	3.677	0.000	1.074
2009	0.742	4.247	–1.104	1.042	0.680	2.255	–0.203	0.982	0.762	3.593	0.000	1.055
2010	0.709	4.330	–1.424	1.075	0.627	2.342	0.137	0.972	0.738	3.628	0.000	1.102
2011	0.821	4.421	–0.898	1.029	0.733	2.280	–0.509	1.003	0.843	3.756	0.000	1.035
2012	0.786	4.354	–0.749	1.027	0.661	2.527	0.073	0.966	0.823	3.552	0.000	1.043
2013	0.764	4.305	–1.093	1.047	0.637	2.616	–0.565	1.013	0.791	3.604	0.000	1.061
2014	0.784	4.140	–1.044	1.051	0.632	2.292	–0.145	0.983	0.815	3.478	0.000	1.062
2015	0.736	3.792	–1.194	1.072	0.579	2.139	–0.026	0.969	0.776	3.127	0.000	1.095
2016	0.781	3.702	–0.980	1.050	0.725	1.964	–0.532	1.010	0.796	3.149	0.000	1.061
2017	0.816	3.343	–0.933	1.041	0.746	1.720	–0.406	0.994	0.834	2.860	0.000	1.055
2018	0.790	3.275	–1.030	1.046	0.726	1.776	–0.745	1.015	0.807	2.775	0.000	1.056
Mean	0.767	4.042	–1.064	1.049	0.658	2.237	–0.334	0.994	0.795	3.382	0.000	1.063

## References

[1] Word Health Organization (WHO) https://www.who.int/health-topics/air-pollution#tab=tab_1.

[2] Liu C, Chen R, Sera F, Vicedo-Cabrera AM, Guo Y, Tong S, Coelho MSZS, Saldiva PHN, Lavigne E, Matus P (2019). Ambient particulate air pollution and daily mortality in 652 cities. N Engl J Med.

[3] Basagaña X, Jacquemin B, Karanasiou A, Ostro B, Querol X, Agis D, Alessandrini E, Alguacil J, Artiñano B, Catrambone M (2015). Short-term effects of particulate matter constituents on daily hospitalizations and mortality in five South-European cities: Results from the MED-PARTICLES project. Environ Int.

[4] Raaschou-Nielsen O, Beelen R, Wang M, Hoek G, Andersen ZJ, Hoffmann B, Stafoggia M, Samoli E, Weinmayr G, Dimakopoulou K (2016). Particulate matter air pollution components and risk for lung cancer. Environ Int.

[5] Lavigne E, Lima I, Hatzopoulou M, Van Ryswyk K, Decou ML, Luo W, van Donkelaar A, Martin RV, Chen H, Stieb DM (2019). Spatial variations in ambient ultrafine particle concentrations and risk of congenital heart defects. Environ Int.

[6] Lavigne E, Donelle J, Hatzopoulou M, Van Ryswyk K, van Donkelaar A, Martin RV, Chen H, Stieb DM, Gasparrini A, Crighton E (2019). Spatiotemporal variations in ambient ultrafine particles and the incidence of childhood asthma. Am J Respir Crit Care Med.

[7] NASA Earth Observations https://neo.sci.gsfc.nasa.gov/view.php?datasetId=MODAL2_M_AER_OD.

[8] Van Donkelaar A, Martin RV, Brauer M, Kahn R, Levy R, Verduzco C, Villeneuve PJ (2010). Global estimates of ambient fine particulate matter concentrations from satellite-based aerosol optical depth: Development and application. Environ Health Perspect.

[9] Koelemeijer R, Homan C, Matthijsen J (2006). Comparison of spatial and temporal variations of aerosol optical thickness and particulate matter over Europe. Atmos Environ.

[10] Gupta P, Christopher SA (2009). Particulate matter air quality assessment using integrated surface, satellite, and meteorological products: Multiple regression approach. J Geophys Res Atmos.

[11] Beckerman BS, Jerrett M, Martin RV, van Donkelaar A, Ross Z, Burnett RT (2013). Application of the deletion/substitution/addition algorithm to selecting land use regression models for interpolating air pollution measurements in California. Atmos Environ.

[12] Vienneau D, de Hoogh K, Beelen R, Fischer P, Hoek G, Briggs D (2010). Comparison of land-use regression models between Great Britain and the Netherlands. Atmos Environ.

[13] De Hoogh K, Héritier H, Stafoggia M, Künzli N, Kloog I (2018). Modelling daily PM_2.5_ concentrations at high spatiotemporal resolution across Switzerland. Environ Pollut.

[14] Kloog I, Koutrakis P, Coull BA, Lee HJ, Schwartz J (2011). Assessing temporally and spatially resolved PM_2.5_ exposures for epidemiological studies using satellite aerosol optical depth measurements. Atmos Environ.

[15] Kloog I, Sorek-Hamer M, Lyapustin A, Coull B, Wang Y, Just AC, Schwartz J, Broday DM (2015). Estimating daily PM_2.5_ and PM_10_ across the complex geo-climate region of Israel using MAIAC satellite-based AOD data. Atmos Environ.

[16] Lee H, Liu Y, Coull B, Schwartz J, Koutrakis P (2011). A novel calibration approach of MODIS AOD data to predict PM_2.5_ concentrations. Atmos Chem Phys.

[17] Stafoggia M, Schwartz J, Badaloni C, Bellander T, Alessandrini E, Cattani G, De’ Donato F, Gaeta A, Leone G, Lyapustin A (2016). Estimation of daily PM_10_ concentrations in Italy (2006-2012) using finely resolved satellite data, land use variables and meteorology. Environ Int.

[18] Chen G, Li S, Knibbs LD, Hamm N, Cao W, Li T, Guo J, Ren H, Abramson MJ, Guo Y (2018). A machine learning method to estimate PM_2.5_ concentrations across China with remote sensing meteorological and land use information. Sci Total Environ.

[19] Di Q, Amini H, Shi L, Kloog I, Silvern R, Kelly J, Sabath MB, Choirat C, Koutrakis P, Lyapustin A (2019). An ensemble-based model of PM_2.5_ concentration across the contiguous United States with high spatiotemporal resolution. Environ Int.

[20] Stafoggia M, Bellander T, Bucci S, Davoli M, de Hoogh K, de’ Donato F, Gariazzo C, Lyapustinf A, Michelozzi P, Renzi M (2019). Estimation of daily PM_10_ and PM_2.5_ concentrations in Italy, 2013-2015, using a spatiotemporal land-use random-forest model. Environ Int.

[21] Yazdi MD, Kuang Z, Dimakopoulou K, Barratt B, Suel E, Amini H, Lyapustin A, Katsouyanni K, Schwartz J (2020). Predicting Fine Particulate Matter (PM_2.5_) in the Greater London Area: An Ensemble Approach using Machine Learning Methods. Remote Sens.

[22] Wei J, Huang W, Li Z, Xue W, Peng Y, Sune L, Cribb M (2019). Estimating 1-km-resolution PM_2.5_ concentrations across China using the space-time random forest approach. Remote Sens Environ.

[23] Chen ZY, Zhang TH, Zhang R, Zhu ZM, Yang J, Chen PY, Ou CQ, Guo Y (2019). Extreme gradient boosting model to estimate PM_2.5_ concentrations with missing-filled satellite data in China. Atmos Environ.

[24] Zhan Y, Luo Y, Deng X, Chen H, Grieneisen ML, Shen X, Zhu L, Zhang M (2017). Spatiotemporal prediction of continuous daily PM_2.5_ concentrations across China using a spatially explicit machine learning algorithm. Atmos Environ.

[25] Polley EC, Rose S, van der Laan MJ, Van der Laan MJ, Rose S (2011). Super Learning. Targeted Learning: Causal Inference for Observational and Experimental Data.

[26] Office for National Statistics (ONS) https://www.ons.gov.uk/peoplepopulationandcommunity/populationandmigration/populationestimates.

[27] Kottek M, Grieser J, Beck C, Rudolf B, Rubel F (2006). World Map of the Köppen-Geiger climate classification updated. Meteorol Z.

[28] Digimap https://digimap.edina.ac.uk/webhelp/os/data_information/os_data_issues/grid_references.htm.

[29] Openair R Package https://cran.r-project.org/web/packages/openair/openair.pdf.

[30] Lyapustin A, Wang Y MCD19A2 MODIS/Terra+Aqua Land Aerosol Optical Depth Daily L2G Global 1km SIN Grid V006. 2018, distributed by NASA EOSDIS Land Processes DAAC.

[31] Bozzo A, Remy S, Benedetti A, Flemming J, Bechtold P, Rodwell MJ, Morcrette JJ (2017). Implementation of a CAMS-Based Aerosol Climatology in the IFSA; European Centre for Medium-Range Weather Forecasts: Reading, UK. https://www.ecmwf.int/sites/default/files/elibrary/2017/17219-implementation-cams-based-aerosol-climatology-ifs.pdf.

[32] European Modelling and Evaluation Programme for the UK (EMEP4UK) http://www.emep4uk.ceh.ac.uk/.

[33] Vieno M, Heal MR, Twigg MM, MacKenzie IA, Braban CF, Lingard JJN, Ritchie S, Beck RC, Móring A, Ots R (2016). The UK particulate matter air pollution episode of March–April 2014: More than Saharan dust. Environ Res Lett.

[34] Vieno M, Dore AJ, Stevenson DS, Doherty R, Heal MR, Reis S, Hallsworth S, Tarrason L, Wind P, Fowler D (2010). Modelling surface ozone during the 2003 heat-wave in the UK. Atmos Chem Phys.

[35] ERA 5 Global Climate Reanalysis https://cds.climate.copernicus.eu/cdsapp#!/dataset/reanalysis-era5-single-levels?tab=overview.

[36] ERA 5 Land Global Climate Reanalysis https://cds.climate.copernicus.eu/cdsapp#!/dataset/reanalysis-era5-land?tab=overview.

[37] UERRA Regional Reanalysis https://cds.climate.copernicus.eu/cdsapp#!/dataset/reanalysis-uerra-europe-soil-levels?tab=overview.

[38] Didan K (2015). MOD13A3 MODIS/Terra Vegetation Indices Monthly L3 Global 1 km SIN Grid V006 [Data set]. NASA EOSDIS LP DAAC.

[39] Copernicus Land Monitoring Service (CLMS) https://land.copernicus.eu/pan-european.

[40] Earth Observation Group (EOG) https://ngdc.noaa.gov/eog/viirs/download_dnb_composites.html.

[41] Ordnance Survey Open Roads https://www.ordnancesurvey.co.uk/documents/os-open-roads-user-guide.pdf.

[42] Civil Aviation Authority (CAA) caa.co.uk/home.

[43] UK Data Service https://www.ukdataservice.ac.uk/.

[44] Schneider dos Santos R (2020). Estimating spatiotemporal air temperature in London (UK) using machine learning and earth observation satellite data. Int J Appl Earth Obs Geoinf.

[45] James G, Witten D, Hastie T, Tibshirani R (2013). An introduction to statistical learning.

[46] Department for Environment, Food & Rural Affairs (DEFRA) Fine Particulate Matter (PM_2.5_) in the UK 2012.

[47] DEFRA. Modelled Background Pollution Data https://uk-air.defra.gov.uk/data/pcm-data.

[48] Savage NH, Agnew P, Davis LS, Ordonez C (2013). Air quality modelling using the Met Office Unified Model (AQUM OS24-26): Model description and initial evaluation. Geosci Model Dev.

[49] Hood C, MacKenzie I, Stocker J, Johnson K, Carruthers D, Vieno M, Doherty R (2018). Air quality simulations for London using a coupled regional-to-local modelling system. Atmos Chem Phys.

[50] Lin C, Heal MR, Vieno M, MacKenzie IA, Armstrong BG, Butland BK, Milojevic A, Chalabi Z, Atkinson RW, Stevenson DS (2017). Spatiotemporal evaluation of EMEP4UK-WRF v4.3 atmospheric chemistry transport simulations of health-related metrics for NO2, O3, PM_10_, and PM_2.5_ for 2001–2010. Geosci Model Dev.

[51] Brookes DM, Stedman JR, Grice SE, Kent AJ, Walker HL, Cooke SL, Vincent KJ, Lingard JJN, Bush TJ, Abbott J (2011). UK Air Quality Modelling under the Air Quality Directive (2008/50/EC) for 2010 Covering the Following Air Quality Pollutants: SO2, NOx, NO2, PM_10_, PM_2.5_, Lead, Benzene, CO, and Ozone. Report for the Department for Environment, Food and Rural Affairs (Defra), Welsh Government, Scottish Government and the Department of the Environment in Northern Ireland. https://uk-air.defra.gov.uk/assets/documents/reports/cat09/1204301513_AQD2010mapsrep_master_v0.pdf.

[52] Air Quality Expert Group (AQEG) Mitigation of United Kingdom PM_2.5_ Concentrations 2013. https://uk-air.defra.gov.uk/assets/documents/reports/cat11/1508060903_DEF-PB14161_Mitigation_of_UK_PM25.pdf.

[53] European Space Agency Copernicus Sentinel-5 Precursor Mission. https://sentinel.esa.int/web/sentinel/missions/sentinel-5p.

[54] European Space Agency. Copernicus Sentinel-4 Mission https://sentinel.esa.int/web/sentinel/missions/sentinel-4.

[55] European Space Agency Copernicus Sentinel-5 Mission. https://sentinel.esa.int/web/sentinel/missions/sentinel-5.

