# Supplementary material for: A Satellite-Based Spatio-Temporal Machine Learning Model to Reconstruct Daily PM2.5 Concentrations across Great Britain
Source: Remote Sens (Basel). Author manuscript; Available in PMC 2021 Jan 5. (PMC7116547; doi:10.3390/rs12223803)
Supplement: Appendix [file EMS104896-supplement-Appendix.pdf]

remote sensing

MDPI

SUPPLEMENTARY MATERIAL

A satellite-based spatio-temporal machine learning model to reconstruct daily $PM_{2.5}$ concentrations across Great Britain

Stage-1: increasing $PM_{2.5}$ measurements using co-located PM_{10} monitors

Table S1. Stage-1 results for 10-Fold CV spatial and temporal domains.

**Stage-1**	Spatial				Temporal			
	R2	RMSE	Inter.	Slope	R2	RMSE	Inter.	Slope
2008	0.489	2.329	3.818	0.660	0.775	3.914	0.000	0.955
2009	0.475	2.433	4.492	0.630	0.865	2.978	0.000	1.008
2010	0.728	2.007	0.814	0.928	0.872	2.831	0.000	0.996
2011	0.784	1.839	0.864	0.932	0.920	2.913	0.000	1.006
2012	0.692	1.959	1.817	0.848	0.920	2.563	0.000	1.007
2013	0.606	2.774	2.757	0.780	0.887	2.876	0.000	1.003
2014	0.691	1.709	1.598	0.864	0.917	2.472	0.000	1.011
2015	0.698	1.496	1.260	0.858	0.899	2.200	0.000	1.003
2016	0.794	1.517	0.146	0.977	0.905	2.190	0.000	1.000
2017	0.824	1.433	0.590	0.921	0.912	2.012	0.000	1.001
2018	0.844	1.155	0.521	0.932	0.897	1.915	0.000	1.009
**Mean**	0.693	1.877	1.698	0.848	0.888	2.624	0.000	1.000

Stage-2: imputing missing satellite-AOD from CAMS modelled-AOD

The Pearson correlation results displayed in Table A2 show a strong correlation across the years between satellite-AOD 0.47μm and the five CAMS modelled-AOD wavelengths. Time 12:00 has been selected to demonstrate the correlation since it is the closest time from both sun-synchronous satellites with near-polar circular orbit to cross the UK territory during daylight from south to north (Aqua) and from north to south (Terra). Table A3 displays the variable importance results from the 0.47μm-RF models measure for the first (2008), middle (2013), and last (2018) years.

Table S2. Pearson correlation between Satellite-AOD 0.47μm and five CAMS modelled-AOD wavelengths at the time 12:00. 0.47μm, 0.55μm, 0.67μm, 0.865μm, and 1.24μm

**Stage-2**	CAMS modelled-AOD				
	0.47μm	0.55μm	0.67μm	0.865μm	1.24μm
2008	0.745	0.734	0.705	0.631	0.439
2009	0.737	0.730	0.711	0.653	0.491
2010	0.706	0.706	0.703	0.681	0.599
2011	0.849	0.843	0.825	0.769	0.614
2012	0.775	0.773	0.762	0.730	0.634
2013	0.814	0.808	0.793	0.750	0.619
2014	0.635	0.598	0.529	0.408	0.227
2015	0.660	0.648	0.616	0.532	0.353
2016	0.761	0.758	0.747	0.707	0.571
2017	0.638	0.635	0.624	0.587	0.484
2018	0.711	0.712	0.705	0.675	0.573

Table S3**.** Relative importance (%) of the predictors in Stage-2.

**Stage 2** –Predictors	2008	2013	2018
CAMS modelled-AOD 0.550μm 12:00	17.18	26.61	25.30
CAMS modelled-AOD 0.469μm 12:00	27.92	25.84	16.74
CAMS modelled-AOD 0.670μm 12:00	4.93	8.95	10.66
Latitude	5.50	3.35	6.70
Longitude	5.95	3.93	6.65
Day of the year	5.15	4.59	4.80
CAMS modelled-AOD 0.865μm 12:00	1.24	1.34	2.04
CAMS modelled-AOD 1.240μm 12:00	1.39	1.42	1.63
CAMS modelled-AOD 0.469μm 15:00	5.64	2.63	1.56
CAMS modelled-AOD 1.240μm 9:00	1.01	1.02	1.23

Note: The top 10 predictors in the RF's importance ranking list are displayed for 2008, 2013, and 2018.

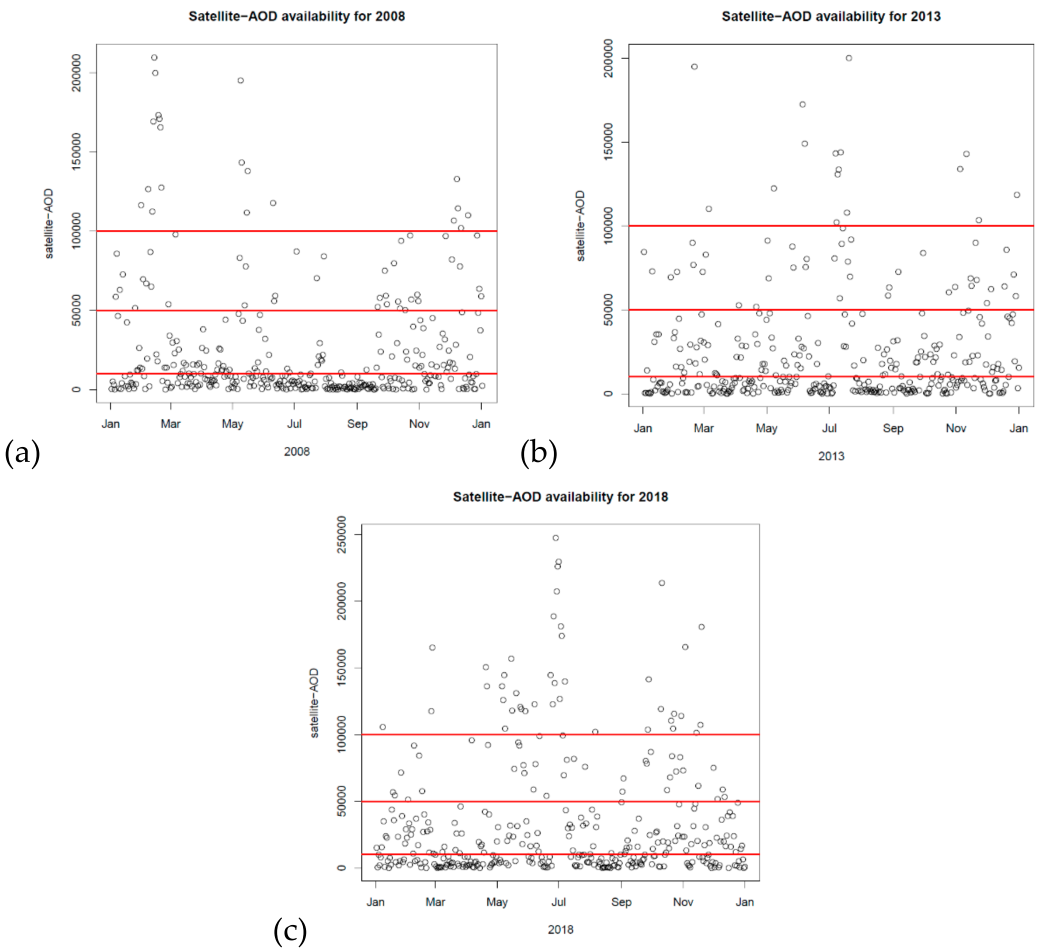

Figure S1. Number of 1 km Great Britain Grid cells with daily satellite-AOD available for (a) 2008, (b) 2013, and (c) 2018. The horizontal red lines represent the thresholds: 10.000, 50.000, and 100.000 1 km grid cells. These data refer to satellite-AOD available after the filtering process to remove bad retrievals.

Stage-3: estimating $PM_{2.5}$ concentrations using spatial and spatio-temporal variables

Table S4. Predicted-$PM_{2.5}$ concentrations obtained from Stage-3 RF models by season were regressed against Stage-1 measured/predicted-$PM_{2.5}$ concentrations in a linear regression model. The CV–R^2 described in three different patterns (overall, spatial, and temporal), RMSE (a measure of the model error, μg/m³), intercept (μg/m³), and slope (μg/m³). In Great Britain, the season is composed of the following months: (i) Winter: December, January, and February, (ii) Spring: March, April, and May, (iii) Summer: June, July, and August, and (iv) Autumn: September, October, and November.

**Stage-3**	Overall		Spatial		Temporal	
**By Season**	R2	RMSE	R2	RMSE	R2	RMSE
Winter	0.818	4.022	0.749	2.029	0.832	3.533
Spring	0.796	3.797	0.704	1.929	0.815	3.286
Summer	0.653	2.749	0.727	1.648	0.602	2.249
Autumn	0.721	3.690	0.704	1.983	0.728	3.163

Stage-4: reconstructing PM2.5 time-series at 1 km grid

Table S5. Distribution of annual averages $PM_{2.5}$ concentrations for all years.

Year	Min	25th Percentile	Mean	Median	75th Percentile	Max	Standard Deviation
2008	4.270	7.160	9.407	9.687	11.366	23.053	2.550
2009	4.200	6.281	8.863	7.922	11.381	20.991	2.920
2010	4.294	6.131	9.229	7.811	13.065	20.649	3.533
2011	3.996	6.090	9.789	8.389	14.119	23.709	4.223
2012	4.117	6.044	9.285	8.064	13.150	24.527	3.622
2013	4.901	6.875	10.172	9.668	13.708	21.554	3.473
2014	5.779	7.757	10.056	9.941	12.235	21.638	2.367
2015	3.488	4.804	7.723	8.219	10.036	21.241	2.705
2016	3.300	4.583	7.304	6.770	9.605	19.048	2.859
2017	3.932	5.491	7.371	6.992	8.878	17.566	2.039
2018	4.142	6.003	8.051	7.823	9.692	18.085	2.061
